# Address of W. H. Monday, M. D., to the Kaufman County Medical Society on His Retiring from the Presidency, at Its Annual Meeting at Kaufman, January 13, 1903

**Published:** 1903-02

**Authors:** 


For Texas Medical Journal.

### Address of W. H. Monday, M. D., to the Kaufman County Medical Society on His Retiring From the Presidency, at Its Annual Meeting at Kaufman, January 13, 1903.

There is strength and wisdom in unity of action. In fact, without it, all bodies, however large, are wanting in power and centralization of power.

We, as physicians of Kaufman county, do not desire to wield any usurpation of power; nor do we desire to deal out injustice or oppression to our patrons. We do not wish to trample under foot the feeble or professionally weak, but we do desire to command that respect which is due to us from those we have to employ us in a professional manner.

There are few, if any, who come in contact with the public who feels more keenly the great responsibility of his duty than the physician. He not only holds the lives of families but the happiness of the household in his hands. Think, for instance, how often it is the case that from bad management, poor judgment, or want of professional ability, orphans are made. Little girls and little boys are left to travel their course through life unprotected and unguided by that gentle parental care they once loved and possessed. This fact alone should be a great incentive to prompt us, as the custodians of the lives, of the health, and of the happiness of the people, to prepare ourselves well to meet the great and mysterious struggles of the physician.

Those of us who will not, can not, or do not, prepare ourselves to walk abreast, from a standpoint of ability, should be looked upon with sympathy; yes, even with scorn. Such a one should be made to realize the sad fact that he has missed his calling, and should be forced to abandon his intention of medicine and seek other environments. Such a man is, perhaps, entitled to more sympathy than is the man who has some little ability but who is wanting in professional and ethical culture.

It is the opinion of the speaker that the most obnoxious character in our ranks is the man who, claiming to be a doctor, resorts to all kind of tricks to decoy the people; who displays and arrogates his work before the people, thus thinking to strengthen himself and belittle his honest and ethical neighbor. I can not commend in terms too strong that it is your duty to look upon such men with distrust; to refuse to meet them, or to recognize or consult with them.

Do your duty as taught by our National and State Code. Remember that if you are an ethical, competent physician, you are an honored man, representing an honorable profession, unexcelled by any calling. Therefore, you are under obligations to your fellow physicians to discharge that duty.

Urge the worthy to join our ranks, and look well to the interests of your local, county and State societies. Every reputable physician in Kaufman county should be a member of this society, and every member of our organization should form himself into a committee of one to see that no one is left unsolicited to join our ranks.

Come out on the side of ethical right, thereby trying to the extent of your ability to elevate your profession. Be on your guard against the unethical man, who, like a roaring lion, tries to demoralize the man who should and does command the respect of the thinking people.

Scientific medicine, and especially scientific surgery and gynecology, has made rapid strides of advancement within the past ten or fifteen years. It is the duty of every man to bestir himself, lest he fall behind. Mix, mingle, consult with and appreciate only the scientific and honorable man, for it is from him also that you will imbibe that spirit of refined culture that constitutes the true physician. It is to the true ethical physician that in time of need and physical disaster the country can look for support and protection. The people are not accurate judges of the competent and incompetent; therefore, it is the duty of the upright and honorable part of the profession to educate them up to this high characteristic standing.

In conclusion, I recommend that this society recognize and elect delegates to the State Association. Give said delegates instructions as to the wishes of the society, and how they shall vote as to the majority or minority report on the constitutional amendment. I also recommend that this society continue holding its meetings quarterly, at some suitable and accessible place, and that every man in this society realize that there is something for him to do individually; realize that there is strength and wisdom in unity of action. Be honest and useful, charitable and prompt, and never neglect the widow or the orphan, and in doing so you will be only second to the angels in heaven.

